# Supplementary material for: Parametric portfolio policy with momentum-based sentiment trading strategy
Source: PLoS One. 2025 Nov 6;20(11):e0335462. doi: 10.1371/journal.pone.0335462 (PMC12591500; doi:10.1371/journal.pone.0335462)
Supplement: S1 Appendix — (DOCX) [file pone.0335462.s001.docx]

**Appendix A**

The sensitivity analysis on parameter $\alpha$, where $\alpha$ varies from 0.0 to 1.0 with an increment of 0.1, is summarized in Table A. The findings indicate that higher values of $\alpha$ place greater weight on the previous period’s portfolio allocation, reducing portfolio rebalancing and consequently lowering turnover rates. Given this trade off, we set $\alpha$ as 0.5 in our main experiments, as it provides a reasonable trade-off between return and turnover rate.

Table A: Sensitivity analysis of parameter $\alpha$ on returns and turnover rate of MV_MS for Dow Jones 30 during the financial crisis

| $\alpha$ | Average return (%) | Std | Sharpe | Turnover |
| --- | --- | --- | --- | --- |
| 0.0 | 0.03 | 11.61 | 0.002 | 1.61 |
| 0.1 | 0.05 | 10.64 | 0.005 | 1.28 |
| 0.2 | 0.16 | 10.55 | 0.015 | 1.18 |
| 0.3 | 0.37 | 10.27 | 0.036 | 1.04 |
| 0.4 | 0.33 | 10.11 | 0.033 | 0.94 |
| 0.5 | 0.72 | 10.01 | 0.072 | 2.94 |
| 0.6 | 0.91 | 10.15 | 0.090 | 0.66 |
| 0.7 | 0.82 | 10.19 | 0.080 | 0.51 |
| 0.8 | 1.05 | 10.04 | 0.105 | 0.36 |
| 0.9 | 1.23 | 10.17 | 0.115 | 0.19 |
| 1.0 | 0.63 | 6.82 | 0.092 | 0 |
