# Supplementary material for: Parametric portfolio policy with momentum-based sentiment trading strategy
Source: PLoS One. 2025 Nov 6;20(11):e0335462. doi: 10.1371/journal.pone.0335462 (PMC12591500; doi:10.1371/journal.pone.0335462)
Supplement: S2 Appendix — (DOCX) [file pone.0335462.s002.docx]

**Appendix B**

To explain the relationship among $\boldsymbol{w}$, $\boldsymbol{w}^{b}$, and $\hat{\boldsymbol{m}}$, we extract $w_{AAPL}$, $w_{AAPL}^{b}$, and $\hat{m}_{\mathrm{AAPL}}$ from $\boldsymbol{w}$, $\boldsymbol{w}^{b}$, and $\hat{\boldsymbol{m}}$, respectively, for the stock AAPL. In Table B, the column Date denotes rebalancing date, while Market value is the performance of the proposed MV_MS model. $s_{AAPL}$ represents the AAPL sentiment score. A higher value of $\theta$ indicates a stronger influence of $\hat{\boldsymbol{m}}$ derived from the momentum-based sentiment strategies. $\hat{\boldsymbol{m}}$ is 0.083 if AAPL is selected as the winner portfolio and 0 otherwise. For example, on June 1, 2009, AAPL is selected into the winner portfolio and $\hat{m}_{AAPL}$ is 0.083. $\theta$ is generated as 1, leading to an increase in $w_{AAPL}$ based on Equation (10). $w_{AAPL}$ decreases while $\theta$ is 0 even though AAPL is selected into winner portfolio on Jun 29, 2009. Thus, $\theta$, parametric portfolio policy matters in terms of portfolio selection.

Table B. The experimental result of AAPL.

| Index | Date | Market value | $\theta$ | ­$w_{AAPL}$ | $w_{AAPL}^{b}$ | $\hat{m}_{AAPL}$ | $s_{AAPL}$ |
| --- | --- | --- | --- | --- | --- | --- | --- |
| 0 | 2007/7/5 | 1,000,000 | 1 | 0.000 | 0 | 0 | 11.33 |
| 1 | 2007/8/2 | 952,511 | 1 | 0.000 | 0 | 0 | 11.03 |
| 2 | 2007/8/30 | 952,911 | 1 | 0.000 | 0 | 0 | 10.05 |
| 3 | 2007/9/28 | 1,018,639 | 1 | 0.000 | 0 | 0 | 10.00 |
| 4 | 2007/10/26 | 1,044,555 | 1 | 0.000 | 0 | 0 | 10.04 |
| 5 | 2007/11/26 | 966,867 | 1 | 0.000 | 0 | 0 | 8.10 |
| 6 | 2007/12/24 | 1,046,136 | 0 | 0.000 | 0 | 0 | 3.19 |
| 7 | 2008/1/24 | 984,721 | 1 | 0.000 | 0 | 0 | 6.69 |
| 8 | 2008/2/22 | 983,564 | 0 | 0.000 | 0 | 0 | 5.50 |
| 9 | 2008/3/24 | 965,806 | 1 | 0.000 | 0 | 0 | 4.93 |
| 10 | 2008/4/21 | 1,005,794 | 0 | 0.000 | 0 | 0 | 5.70 |
| 11 | 2008/5/19 | 1,045,716 | 0 | 0.000 | 0 | 0 | 8.13 |
| 12 | 2008/6/17 | 1,032,435 | 1 | 0.000 | 0 | 0 | 11.18 |
| 13 | 2008/7/16 | 952,429 | 1 | 0.000 | 0 | 0 | 9.32 |
| 14 | 2008/8/13 | 1,014,078 | 0 | 0.000 | 0 | 0 | 8.60 |
| 15 | 2008/9/11 | 959,870 | 1 | 0.000 | 0 | 0 | 7.84 |
| 16 | 2008/10/9 | 659,098 | 1 | 0.000 | 0 | 0 | 8.02 |
| 17 | 2008/11/6 | 653,058 | 0 | 0.000 | 0 | 0 | 11.67 |
| 18 | 2008/12/5 | 631,037 | 1 | 0.000 | 0 | 0 | 9.35 |
| 19 | 2009/1/6 | 681,415 | 1 | 0.000 | 0 | 0 | 10.06 |
| 20 | 2009/2/4 | 610,368 | 1 | 0.000 | 0 | 0 | 11.02 |
| 21 | 2009/3/5 | 509,608 | 1 | 0.000 | 0 | 0 | 12.00 |
| 22 | 2009/4/2 | 683,472 | 0 | 0.000 | 0 | 0 | 15.96 |
| 23 | 2009/5/1 | 771,252 | 0 | 0.000 | 0 | 0 | 11.43 |
| 24 | 2009/6/1 | 810,970 | 1 | 0.042 | 0 | 0.083 | 12.51 |
| 25 | 2009/6/29 | 797,919 | 0 | 0.022 | 0 | 0.083 | 13.44 |
| 26 | 2009/7/28 | 857,523 | 0 | 0.011 | 0 | 0 | 11.80 |
| 27 | 2009/8/25 | 889,824 | 0 | 0.016 | 0 | 0 | 11.80 |
| 28 | 2009/9/23 | 960,026 | 1 | 0.018 | 0 | 0 | 9.78 |
| 29 | 2009/10/21 | 956,809 | 1 | 0.020 | 0 | 0 | 6.90 |
| 30 | 2009/11/18 | 983,066 | 1 | 0.020 | 0 | 0 | 4.05 |
