# Supplementary material for: Parametric portfolio policy with momentum-based sentiment trading strategy
Source: PLoS One. 2025 Nov 6;20(11):e0335462. doi: 10.1371/journal.pone.0335462 (PMC12591500; doi:10.1371/journal.pone.0335462)
Supplement: Attachment — Submitted filename: Final version_Response letter_09222025.docx [file pone.0335462.s005.docx]

**Reply to the Editor and Reviewers’ Comments**

Journal: PLOS ONE

Ref. No.: PONE-D-25-34570
Title: Parametric portfolio policy with momentum-based sentiment trading strategy

**Editor**:

1. The methodological framework has not been validated through current real-world

case studies or robust alternative methods.

**Response**:

Thank you for your feedback. We have added Apple as an example to illustrate the parametric portfolio policy, which incorporates a momentum-based sentiment trading strategy in Appendix B. Buy and hold and naïve strategies have also been added for comparisons in Tables 1-4.

2. Moreover, outdated references and insufficient empirical results diminish the

strength of your findings.

**Response**:

We have incorporated six recent references, including two from PLOS One, to support the use of a parametric portfolio policy combined with a momentum-based sentiment strategy to enhance asset allocation.

3. Additionally, discrepancies in your experimental design, particularly the

inconsistency in asset selection thresholds, lack of sensitivity analyses (e.g., parameter alpha), and insufficient explanations of key methodological details (such as sentiment computation and mapping news to specific stocks) severely undermine the clarity and reliability of your findings

**Response**: We have added parameter alpha sensitivity analyses in Appendix A and explained the sentiment computation, using APPLE as an example in Appendix B.

4. Please remove any funding-related text from the manuscript and let us know how you would like to update your Funding Statement. Currently, your Funding Statement reads as follows:

 [The authors acknowledge the support provided by the National Science and Technology Council, Taiwan (Grant number: NSTC 111-2410-H-141-004-).]

Please include your amended statements within your cover letter; we will change the online submission form on your behalf.

**Response**: We have removed the funding-related text from the manuscript and included the amended statements in the cover letter.

**Reviewer #1**:

1. The paper proposes a method but fails to implement it to some read-world case

study.

**Response**:

We have presented the experimental results of the MV_MS model and taken Apple Inc. (AAPL), a composite stock within the S&P500 Index, from July 5, 2007, to November 18, 2009, as an example in Appendix A. The decision variable $\theta$ governs the trade-off between $\boldsymbol{w}^{b}$ and $\hat{\boldsymbol{m}}$ in Eq. (10).

$\boldsymbol{w}=\boldsymbol{w}^{h}+\left( \left( 1-\theta\right)\boldsymbol{w}^{b}+\theta\hat{\boldsymbol{m}} \right)+\boldsymbol{l}^{+}-\boldsymbol{l}^{-},$	(10)

where the holding weights ($\boldsymbol{w}^{h}$) are from the previous period and the benchmark weights ($\boldsymbol{w}^{b}$) are generated from the benchmark mean-variance model. The characteristic vector $\hat{\boldsymbol{m}}$ is derived from either the momentum or the momentum-based sentiment strategies.

To better explain the relationship between $\boldsymbol{w}$, $\boldsymbol{w}^{b}$, and $\hat{\boldsymbol{m}}$, we extract $w_{AAPL}$, $w_{AAPL}^{b}$, and $\hat{m}_{\mathrm{AAPL}}$ from $\boldsymbol{w}$, $\boldsymbol{w}^{b}$, and $\hat{\boldsymbol{m}}$, respectively, for the stock AAPL. In Table B, the column *Date* denotes rebalancing date, while *Market Value* is the performance of the proposed MV_MS model. A higher value of $\theta$ indicates a stronger influence of $\hat{\boldsymbol{m}}$ derived from the momentum-based sentiment strategies. $\hat{\boldsymbol{m}}$ is 0.083 if AAPL is selected as the winner portfolio and 0 otherwise. For example, on June 1, 2009, AAPL is selected into the winner portfolio and $\hat{m}_{AAPL}$ is 0.083. $\theta$ is generated as 1, leading to an increase in $w_{AAPL}$ based on Equation (10). $w_{AAPL}$ decreases while $\theta$ is 0 even though AAPL is selected into winner portfolio on Jun 29, 2009. Thus, $\theta$, parametric portfolio policy matters in terms of portfolio selection. $s_{AAPL}$ represents the AAPL sentiment score.

Table B. The experimental result of AAPL.

| Index | Date | Market value | $\theta$ | ­$w_{AAPL}$ | $w_{AAPL}^{b}$ | $\hat{m}_{AAPL}$ | $s_{AAPL}$ |
| --- | --- | --- | --- | --- | --- | --- | --- |
| 0 | 2007/7/5 | 1,000,000 | 1 | 0.000 | 0 | 0 | 11.33 |
| 1 | 2007/8/2 | 952,511 | 1 | 0.000 | 0 | 0 | 11.03 |
| 2 | 2007/8/30 | 952,911 | 1 | 0.000 | 0 | 0 | 10.05 |
| 3 | 2007/9/28 | 1,018,639 | 1 | 0.000 | 0 | 0 | 10.00 |
| 4 | 2007/10/26 | 1,044,555 | 1 | 0.000 | 0 | 0 | 10.04 |
| 5 | 2007/11/26 | 966,867 | 1 | 0.000 | 0 | 0 | 8.10 |
| 6 | 2007/12/24 | 1,046,136 | 0 | 0.000 | 0 | 0 | 3.19 |
| 7 | 2008/1/24 | 984,721 | 1 | 0.000 | 0 | 0 | 6.69 |
| 8 | 2008/2/22 | 983,564 | 0 | 0.000 | 0 | 0 | 5.50 |
| 9 | 2008/3/24 | 965,806 | 1 | 0.000 | 0 | 0 | 4.93 |
| 10 | 2008/4/21 | 1,005,794 | 0 | 0.000 | 0 | 0 | 5.70 |
| 11 | 2008/5/19 | 1,045,716 | 0 | 0.000 | 0 | 0 | 8.13 |
| 12 | 2008/6/17 | 1,032,435 | 1 | 0.000 | 0 | 0 | 11.18 |
| 13 | 2008/7/16 | 952,429 | 1 | 0.000 | 0 | 0 | 9.32 |
| 14 | 2008/8/13 | 1,014,078 | 0 | 0.000 | 0 | 0 | 8.60 |
| 15 | 2008/9/11 | 959,870 | 1 | 0.000 | 0 | 0 | 7.84 |
| 16 | 2008/10/9 | 659,098 | 1 | 0.000 | 0 | 0 | 8.02 |
| 17 | 2008/11/6 | 653,058 | 0 | 0.000 | 0 | 0 | 11.67 |
| 18 | 2008/12/5 | 631,037 | 1 | 0.000 | 0 | 0 | 9.35 |
| 19 | 2009/1/6 | 681,415 | 1 | 0.000 | 0 | 0 | 10.06 |
| 20 | 2009/2/4 | 610,368 | 1 | 0.000 | 0 | 0 | 11.02 |
| 21 | 2009/3/5 | 509,608 | 1 | 0.000 | 0 | 0 | 12.00 |
| 22 | 2009/4/2 | 683,472 | 0 | 0.000 | 0 | 0 | 15.96 |
| 23 | 2009/5/1 | 771,252 | 0 | 0.000 | 0 | 0 | 11.43 |
| 24 | 2009/6/1 | 810,970 | 1 | 0.042 | 0 | 0.083 | 12.51 |
| 25 | 2009/6/29 | 797,919 | 0 | 0.022 | 0 | 0.083 | 13.44 |
| 26 | 2009/7/28 | 857,523 | 0 | 0.011 | 0 | 0 | 11.80 |
| 27 | 2009/8/25 | 889,824 | 0 | 0.016 | 0 | 0 | 11.80 |
| 28 | 2009/9/23 | 960,026 | 1 | 0.018 | 0 | 0 | 9.78 |
| 29 | 2009/10/21 | 956,809 | 1 | 0.020 | 0 | 0 | 6.90 |
| 30 | 2009/11/18 | 983,066 | 1 | 0.020 | 0 | 0 | 4.05 |

2. The data used are old, they need to update the references and expand their results.

**Response**: We have added six references to address the importance using parametric portfolio policy to portfolio selection. Recent advancements in portfolio research highlight the versatility of parametric portfolio policies in improving asset allocation outcomes. Zeng et al. (2025) found that generalized linear models enhance momentum strategies by integrating firm characteristics to capture return persistence more effectively. Golosnoy et al. (2025) demonstrated that combining competing prediction rules within a parametric structure enhances the stability of global minimum variance portfolios, particularly by detecting risky assets. Beyond linear forms, Li et al. (2025) proposed nonlinear neural network–based policies that directly map firm characteristics into portfolio weights, while applying heuristic smoothing to control turnover and transaction costs. Arasteh (2025) added a multi-period dimension by incorporating real options and ARIMA–GARCH forecasts, highlighting the adaptability of parametric policies under uncertainty. These studies demonstrate that parametric portfolio policies, whether linear, nonlinear, or dynamic, provide a coherent framework for improving predictive accuracy and economic feasibility in portfolio optimization.

Arasteh, A. (2025). A data-driven prediction method for multi-period portfolio optimization using the real options approach. *Finance Research Letters*, *80*, 107403.

Golosnoy, V., Gribisch, B., Schmid, W., & Seifert, M. I. (2025). Combining portfolio rules to improve prediction of global minimum variance portfolio weights. *The European Journal of Finance*, 1-18.

Hasan, M., Ahmed, T., Islam, M. R., & Uddin, M. P. (2024). Leveraging textual information for social media news categorization and sentiment analysis. *Plos one*, *19*(7), e0307027.

Li, H., Wu, C., & Zhou, C. (2025). Machine+ Heuristics: Nonlinear parametric portfolio policies with economic restrictions. Journal of Financial Markets, 101001.

Usmani, S., & Shamsi, J. A. (2023). LSTM based stock prediction using weighted and categorized financial news. *PloS one*, *18*(3), e0282234.

Zeng, H., Marshall, B. R., Nguyen, N. H., & Visaltanachoti, N. (2025). Improving momentum returns using generalized linear models. *International Review of Finance*, *25*(2), e70014.

3. The authors also need to validate their results using some alternative methods in order to show the effectiveness of their results.

**Response**: To validate the results, we have employed two additional alternative benchmarks: naïve and buy-and-hold strategies. The results are reported in Tables 1–4. Across both the S&P 500 and Dow Jones 30 datasets, the proposed MV_MS model consistently outperformed the benchmark in terms of realized return, Sharpe ratio, and market value (Tables 1 and 3). During the financial crisis (2007–2009), MV_MS maintained positive returns and superior Sharpe ratios compared to both Naïve and buy-and-hold benchmarks. From Tables 2 and 4, the momentum or momentum-based sentiment strategy (MV_M and MV_MS) incurs higher trade costs due to more frequent adjustments in asset allocation, resulting in a higher turnover ratio. The findings confirm that while conventional benchmarks provide simplicity and cost efficiency, the proposed MV_MS model delivers more robust and effective portfolio performance, particularly under volatile conditions. The additional validation demonstrates the higher practical value of MV_MS compared to conventional mean-variance models (MV, MV_M) and naïve and buy-and-hold strategies.

Table 1. Portfolio comparison using the S&P 500 and the Dow Jones 30 datasets (January 1, 2005 to January 4, 2022).

|  | Realized | | | Market value | |
| --- | --- | --- | --- | --- | --- |
|  | Average  Return (%) | Standard deviation (%) | Sharpe ratio | Final | Average |
| Panel A: S&P 500 |  |  |  |  |  |
| MV | 0.94% | 5.30% | 0.177 | 5,145,086 | 2,100,427 |
| MV_M | 1.05% | 5.18% | 0.203 | 6,597,270 | 2,696,578 |
| MV_MS | 1.29% | 5.19% | 0.249 | 10,788,043 | 3,713,499 |
| Buy and hold | 1.06% | 4.94% | 0.215 | 7,686,905 | 3,427,768 |
| Naïve | 1.02% | 5.05% | 0.203 | 6,988,687 | 3,123,605 |
| S&P 500 index (S&P500) | 0.04% | 1.23% | 0.033 |  |  |
| Panel B: Dow Jones 30 |  |  |  |  |  |
| MV | 0.95% | 4.09% | 0.232 | 6,003,082 | 2,657,274 |
| MV_M | 1.13% | 4.23% | 0.267 | 8,575,916 | 3,511,711 |
| MV_MS | 1.24% | 4.57% | 0.272 | 10,486,295 | 4,000,107 |
| Buy and hold | 1.15% | 4.90% | 0.234 | 9,318,815 | 3,698,810 |
| Naïve | 0.92% | 4.37% | 0.210 | 2,883,092 | 5,981,745 |
| Dow Jones 30 index (DJ30) | 0.03% | 1.17% | 0.031 |  |  |

Table 2. Comparing trade numbers and cost using the S&P 500 and the Dow Jones 30 datasets (January 1, 2005 to January 4, 2022).

|  | Avg. number of buying assets | Avg. trading  cost ($) | Accum. trading  costs ($) | Avg. turnover rate (%) |
| --- | --- | --- | --- | --- |
| Panel A: S&P 500 |  |  |  |  |
| MV | 13 | 569 | 118,819 | 0.04% |
| MV_M | 56 | 5,697 | 1,190,600 | 0.10% |
| MV_MS | 48 | 5,911 | 1,235,323 | 0.15% |
| Buy and hold | 382 | 11 | 2,494 | 0% |
| Naïve | 382 | 455 | 100,202 | 0% |
| Panel B: Dow Jones 30 |  |  |  |  |
| MV | 8 | 299 | 62,547 | 0.33% |
| MV_M | 17 | 1,065 | 222,666 | 1.47% |
| MV_MS | 14 | 1,172 | 245,013 | 2.39% |
| Buy and hold | 27 | 11 | 2,494 | 0% |
| Naïve | 27 | 309 | 67,923 | 0% |

**Avg. means average. Accum. means accumulated.*

Table 3. Portfolio comparisons using the S&P 500 and the Dow Jones 30 datasets (from Jan. 10, 2007, to Dec. 30, 2009).

|  | Realized | | | Market value | |
| --- | --- | --- | --- | --- | --- |
|  | Average  Return (%) | Standard deviation (%) | Sharpe ratio | Final | Average |
| Panel A: S&P 500 |  |  |  |  |  |
| MV | -0.26% | 7.28% | -0.035 | 849,903 | 889,783 |
| MV_M | 0.51% | 9.76% | 0.051 | 996,218 | 961,941 |
| MV_MS | 0.51% | 10.54% | 0.048 | 983,066 | 883,273 |
| Buy and hold | -0.28% | 8.50% | -0.033 | 816,174 | 818,060 |
| Naïve | 0.15% | 0.67% | 0.015 | 899,983 | 835,037 |
| S&P 500 index (S&P500) | -0.01% | 1.89% | -0.006 |  |  |
| Panel B: Dow Jones 30 |  |  |  |  |  |
| MV | -0.12% | 9.08% | -0.013 | 842,448 | 843,541 |
| MV_M | -0.37% | 9.37% | -0.039 | 775,966 | 815,582 |
| MV_MS | 0.72% | 10.01% | 0.072 | 1,061,853 | 941,796 |
| Buy and hold | 0.07% | 8.13% | 0.009 | 917,770 | 866,802 |
| Naïve | 0.31% | 8.68% | 0.035 | 970,089 | 879,293 |
| Dow Jones 30 index (DJ30) | 0.01% | 1.73% | -0.004 |  |  |

Table 4. Comparing trade numbers and costs using the S&P 500 and the Dow Jones 30 datasets during the financial crisis (from January 10, 2007 to December 30, 2009).

|  | Avg. number of buying assets | Avg. trading  cost ($) | Accum. trading  costs ($) | Avg. turnover rate (%) |
| --- | --- | --- | --- | --- |
| Panel A: S&P 500 |  |  |  |  |
| MV | 11 | 370 | 11,479 | 0.05% |
| MV_M | 51 | 1,668 | 51,727 | 0.11% |
| MV_MS | 36 | 783 | 24,287 | 0.19% |
| Buy and hold | 382 | 80 | 2,494 | 0% |
| Naïve | 382 | 234 | 7,260 | 0% |
| Panel B: Dow Jones 30 |  |  |  |  |
| MV | 4 | 809 | 25,075 | 1.54% |
| MV_M | 12 | 205 | 6,361 | 2.08% |
| MV_MS | 13 | 301 | 9,340 | 2.94% |
| Buy and hold | 27 | 80 | 2,494 | 0% |
| Naïve | 27 | 198 | 6,151 | 0% |

**Reviewer #2**:

**Comment 1**: In the proposed model, the top 30% of assets are first selected based on past returns, followed by an additional filtering of the top 10% with the most positive sentiment. This implies that only the top 3% of assets are finally selected.
On the other hand, in MV_M, the top 10% assets are selected solely based on returns.
This discrepancy in the number of selected stocks makes the comparison between MV_M and MV_MS potentially imbalanced.
It would strengthen the analysis to either (a) tune the 30% and 10% thresholds, or (b) compare both models using the same number of selected stocks.
**Response**: Initially, both MV_M and MV_MS select assets within the top 30% in terms of return. Subsequently, MV_MS filters these by selecting the top 10% of assets with the most positive sentiment.

**Comment 2**: The parameter alpha, which controls the balance between the current and previous portfolio weights, is fixed at 0.5.
It would be beneficial to include a sensitivity analysis varying alpha to assess its impact on both turnover and portfolio performance.
Comparing turnover rates and returns under different alpha values could add depth to the empirical results.
**Response**: The linear combination of target ($\boldsymbol{w}^{target}$) and holding portfolios represents the portfolio weights ($\boldsymbol{w}$), as shown in Eq. (7). The holding portfolio weight ($\boldsymbol{w}^{h}) \mathrm{is}$the previous investments and is, therefore, regarded as the weight before rebalancing.

$\boldsymbol{w}=\alpha\boldsymbol{w}^{h}+\left( 1-\alpha\right)\boldsymbol{w}^{target}$,	(7)

where $\alpha$ is the constant, ranging from 0 to 1, which represents the trade-off between the holding and target portfolios in the static model. $\boldsymbol{w}$ represents the rebalanced portfolio weight vector, which is derived by integrating the holding and target weight vectors.

The sensitivity analysis on parameter $\alpha$, where $\alpha$ varies from 0.0 to 1.0 with an increment of 0.1, is summarized in Table A. The findings indicate that higher values of $\alpha$ place greater weight on the previous period’s portfolio allocation, reducing portfolio rebalancing and consequently lowering turnover rates. Given this trade off, we set $\alpha$ as 0.5 in our main experiments, as it provides a reasonable trade-off between return and turnover rate.

Table A: Sensitivity analysis of parameter $\alpha$ on returns and turnover rate of MV_MS for Dow Jones 30 during the financial crisis

| $\alpha$ | Average return (%) | Std | Sharpe | Turnover |
| --- | --- | --- | --- | --- |
| 0.0 | 0.03 | 11.61 | 0.002 | 1.61 |
| 0.1 | 0.05 | 10.64 | 0.005 | 1.28 |
| 0.2 | 0.16 | 10.55 | 0.015 | 1.18 |
| 0.3 | 0.37 | 10.27 | 0.036 | 1.04 |
| 0.4 | 0.33 | 10.11 | 0.033 | 0.94 |
| 0.5 | 0.72 | 10.01 | 0.072 | 2.94 |
| 0.6 | 0.91 | 10.15 | 0.090 | 0.66 |
| 0.7 | 0.82 | 10.19 | 0.080 | 0.51 |
| 0.8 | 1.05 | 10.04 | 0.105 | 0.36 |
| 0.9 | 1.23 | 10.17 | 0.115 | 0.19 |
| 1.0 | 0.63 | 6.82 | 0.092 | 0 |

**Comment 3**: Please double-check the sign of the last term in Equation (8).
Should it be a plus sign rather than a minus?
**Response**: Thank you very much for the correction.

**Comment 4**: The variables l+ and l- appear to represent the quantities of buying and selling, respectively, between consecutive rebalancing periods.
However, this explanation is not clearly articulated in the manuscript.
Adding a concise but clear explanation will help readers better understand the role of these terms in calculating transaction costs.
**Response**: Thank you for your feedback. We have expanded the description as follows:

$\boldsymbol{l}^{+}$ and $\boldsymbol{l}^{-}$ in Eq. (10) indicate the dynamic buying and selling propositions for each rebalancing. For example, $\boldsymbol{l}^{+}=\left[ l_{1}^{+},l_{2}^{+},\ldots,l_{n}^{+} \right]^{T}$ and $\boldsymbol{l}^{-}=\left[ l_{1}^{-},l_{2}^{-},\ldots,l_{n}^{-} \right]^{T}$denotes the weight vectors of buying and selling, where $l_{i}^{+}$ and $l_{i}^{-}$represent the buying and selling weights of the *i*-th stock, respectively. To illustrate, $l_{2}^{+}$=0.02 indicates that 2% of the budget is allocated to purchase the second stock, which incurs a trade cost of ­$p_{1}l_{2}^{+}$. Furthermore, Equation (9) indicates that the budget is equal to 1, considering the summation portfolio weight ($\boldsymbol{1}^{T}\boldsymbol{w}$) and corresponding trade costs ($p_{1}\boldsymbol{1}^{T}\boldsymbol{l}^{+}+p_{2}\boldsymbol{1}^{T}\boldsymbol{l}^{-}$).

$$\boldsymbol{1}^{T}\boldsymbol{w}+p_{1}\boldsymbol{1}^{T}\boldsymbol{l}^{+}+p_{2}\boldsymbol{1}^{T}\boldsymbol{l}^{-}=1$$

(9)

**Comment 5**: The description of sentiment score computation is overly brief. Simply stating that NLTK was used is insufficient.
More detail is needed regarding how the sentiment scores were extracted from the text, such as which lexicon or model was applied, how documents were tokenized, and how firm-level sentiment was aggregated.
**Response**: The revised manuscript includes detailed explanation of our text mining process for mapping news articles to specific stocks. The process comprises the following three steps:

Step 1. Data collection: Using the company name keywords listed in Table C, we retrieved articles from The Financial Times that contained any of these keywords. The data collection period spanned from January 1, 2005, to June 30, 2022. To ensure consistency, we retained only the 413 companies that remained continuously listed in the S&P 500 during the entire period. This process resulted in a total of 796,947 articles.

Table C. Example symbols.

| Ticker Symbol | Company name 1 | Company name 2 |
| --- | --- | --- |
| AOS | A. O. Smith | A. O. Smith Corporation |
| ABMD | ABIOMED | ABIOMED Inc. |
| AME | AMETEK | AMETEK Inc. |
| ... | ... | ... |
| ZBRA | Zebra Technologies |  |
| ZBH | Zimmer Biomet Holdings |  |
| ZTS | Zoetis |  |

Step 2. Text preprocessing and sentiment scoring: We first applied stopword removal to eliminate non-informative terms and converted all words to lowercase for consistency. Next, we used the Sentiment Intensity Analyzer from the valence aware dictionary and sentiment reasoner (VADER) library in NLTK to assign a sentiment label to each article: 1 (positive), 0 (neutral), or −1 (negative).

For example, we analyze the sentiment of the article “Web groups battle for pennies from customers.” The article details are shown in Table D. “Query” indicates the article’s keyword. Since the article contains 2,203 characters, we present the crucial content related to Apple in Table D.

Table D News article details “Web groups battle for pennies from customers.”

| Query | Apple |
| --- | --- |
| Title | Web groups battle for pennies from customers |
| Date | 2009-05-02 |
| Url | https://www.ft.com/content/a29b81f0-3675-11de... |
| Content | A diverse set of companies is doing big business selling cheap online goods and services across the web. **Apple** has sold more than 6bn songs for $1 apiece on iTunes…**Apple** introduced the iTunes store and Skype began charging pennies for online calling. This softened consumer resistance, opening the door for a generation of online games and applications to levy similar charges. The reason we buy things in the real world is it makes us look and feel good. That’s moving online,” Vikas Gupta, head of Jambool, a payment system for applications and games, said. |

We processed the article using the Sentiment Intensity Analyzer from the NLTK package. The polarity_scores function outputs the following dictionary: {'neg': 0.043, 'neu': 0.892, 'pos': 0.065, 'compound': 0.821}. Here, neg represents the negative sentiment score, neu is the neutral score, pos is the positive score, and compound is a normalized aggregated score (Hutto and Gilbert, 2014). According to Du et al. (2024), VADER is widely applied in financial sentiment analysis.

We use the compound score, which reflects the overall sentiment by incorporating both positive and negative components, as the sentiment measure of this article. Since the compound score is greater than zero, the article is labeled as 1 (positive sentiment).

Step 3. Aggregating sentiment and generating the characteristic vector ($\hat{\boldsymbol{m}}$). If the company is mentioned in several news articles, we average the labels. While the average score is higher than 0, we identify the company sentiment as 1 (positive) on that day. We identify the sentiment of the company as −1 (negative) if the average score is lower than 0. For instance, we have five articles related to AAPL on May 02, 2009. The articles’ compound sentiment scores and labels are provided in Table E.

Table E. News article details related to AAPL on May 02, 2009.

| ID | Query | Title | Content | Compound sentiment score | Label |
| --- | --- | --- | --- | --- | --- |
| 731203 | Apple | Twitter’s high-water mark of hype… | If you thought the hype surrounding Twitter ha... | 0.9546 | 1 |
| 731206 | Apple | The small fortune to be made on the web | On Pet Society, one of the most popular social... | 0.996 | 1 |
| 731208 | Apple | Web groups battle for pennies from customers | A diverse set of companies is doing big busine... | 0.821 | 1 |
| 731210 | Apple | Bulls take the market by the horns | The bulls appear to have the upper hand.\nSinc... | 0.9794 | 1 |
| 1731212 | Apple | Genesis of the debt disaster | In the 1990s, a young team at Wall Street inve... | 0.9977 | 1 |

Based on the articles’ labels in Table E, the average score of AAPL at May 02, 2009 is 1. The instances of AAPL labels are shown in Table F.

Table F. AAPL sentiment

| Date | Compound sentiment score | Label | Sum of Label |
| --- | --- | --- | --- |
| 2009-05-02 | 0.9911 | 1 | 13 |
| … | … | … | … |
| 2009-06-01 | None | None | 13 |

In Table F, the sum of Label indicates the sum of the label from the current date to the previous 180 calendar days. Since it is possible that companies have no news on some days, especially for small companies, we use the label sum to determine companies’ sentiment. $\hat{\boldsymbol{m}}$ of MV_MS considering both the historical return and sentiment is provided in Table G.

Table G. Elements’ value of $\hat{\boldsymbol{m}}$ for MV_MS in the S&P 500 during the financial crisis.

|  | A | AAP | AAPL | ABC | ABT | ACN | ADBE | ADI | ADM |
| --- | --- | --- | --- | --- | --- | --- | --- | --- | --- |
| 2007/7/5 | 0 | 0 | 0 | 0 | 0 | 0 | 0 | 0 | 0 |
| 2007/8/2 | 0 | 0 | 0 | 0 | 0 | 0 | 0 | 0 | 0 |
| 2007/8/30 | 0 | 0 | 0 | 0 | 0 | 0 | 0 | 0 | 0 |
| 2007/9/28 | 0 | 0 | 0 | 0 | 0 | 0 | 0 | 0 | 0 |
| 2007/10/26 | 0 | 0 | 0 | 0 | 0 | 0 | 0 | 0 | 0 |
| 2007/11/26 | 0 | 0 | 0 | 0 | 0 | 0 | 0 | 0 | 0 |
| 2007/12/24 | 0 | 0 | 0 | 0 | 0 | 0 | 0 | 0 | 0 |
| 2008/1/24 | 0 | 0 | 0 | 0 | 0 | 0 | 0 | 0 | 0 |
| 2008/2/22 | 0 | 0 | 0 | 0 | 0 | 0.083 | 0 | 0 | 0 |
| 2008/3/24 | 0 | 0 | 0 | 0 | 0 | 0.083 | 0 | 0 | 0 |
| 2008/4/21 | 0 | 0 | 0 | 0 | 0 | 0 | 0 | 0 | 0 |
| 2008/5/19 | 0 | 0 | 0 | 0 | 0 | 0 | 0 | 0 | 0 |
| 2008/6/17 | 0 | 0 | 0 | 0 | 0 | 0 | 0 | 0 | 0 |
| 2008/7/16 | 0 | 0 | 0 | 0 | 0 | 0 | 0 | 0.083 | 0 |
| 2008/8/13 | 0 | 0.083 | 0 | 0 | 0 | 0 | 0 | 0.083 | 0 |
| 2008/9/11 | 0 | 0 | 0 | 0 | 0 | 0 | 0.083 | 0 | 0 |
| 2008/10/9 | 0 | 0 | 0 | 0 | 0 | 0 | 0 | 0 | 0 |
| 2008/11/6 | 0 | 0 | 0 | 0 | 0 | 0 | 0 | 0 | 0 |
| 2008/12/5 | 0 | 0 | 0 | 0 | 0 | 0 | 0 | 0 | 0 |
| 2009/1/6 | 0 | 0 | 0 | 0 | 0 | 0 | 0 | 0 | 0 |
| 2009/2/4 | 0 | 0 | 0 | 0 | 0 | 0 | 0 | 0 | 0 |
| 2009/3/5 | 0 | 0 | 0 | 0 | 0 | 0.083 | 0 | 0 | 0 |
| 2009/4/2 | 0 | 0 | 0 | 0 | 0 | 0 | 0 | 0 | 0 |
| 2009/5/1 | 0 | 0 | 0 | 0 | 0 | 0 | 0 | 0 | 0 |
| 2009/6/1 | 0 | 0 | 0.083 | 0 | 0 | 0 | 0 | 0 | 0 |
| 2009/6/29 | 0 | 0.083 | 0.083 | 0 | 0 | 0 | 0 | 0 | 0 |
| 2009/7/28 | 0 | 0 | 0 | 0 | 0 | 0 | 0 | 0 | 0 |
| 2009/8/25 | 0 | 0 | 0 | 0 | 0 | 0 | 0 | 0 | 0 |
| 2009/9/23 | 0 | 0 | 0 | 0 | 0 | 0 | 0 | 0 | 0 |
| 2009/10/21 | 0 | 0 | 0 | 0 | 0 | 0 | 0 | 0 | 0 |
| 2009/11/18 | 0 | 0 | 0 | 0 | 0 | 0 | 0 | 0 | 0 |

References

Hutto, C., & Gilbert, E. (2014, May). *VADER: A parsimonious rule-based model for sentiment analysis of social media text*. In *Proceedings of the International AAAI Conference on Web and Social Media* (Vol. 8, No. 1, pp. 216–225). Du, K., Xing, F., Mao, R., & Cambria, E. (2024). Financial sentiment analysis: Techniques and applications. *ACM Computing Surveys*, *56*(9), 1-42.

**Comment 6**: Please consider including descriptive statistics for the news data used in the sentiment analysis.
This would help validate the robustness and distribution of the sentiment signals.
**Response**: The revised manuscript includes descriptive statistics for the news dataset used in the sentiment analysis (Table G). The dataset contains a total of 796,947 news articles covering 488 companies from January 1, 2005, to December 31, 2022 (6,574 trading days). At the company level, the maximum number of articles is 46,672, with a minimum of 1, an average of 1,633 articles per company, and a standard deviation of 4,504. These statistics demonstrate the breadth of coverage and the variability of news distribution across companies, supporting the robustness of the sentiment signals derived from the dataset.

Table G. Descriptive statistics of the news dataset used in sentiment analysis

| Statistic | Value |
| --- | --- |
| Total number of articles | 796,947 |
| Number of companies | 488 |
| Maximum articles (per firm) | 46,672 |
| Minimum articles (per firm) | 1 |
| Average articles (per firm) | 1,633 |
| Standard deviation (per firm) | 4,504 |
| Trading days covered | 6,574 |
| Sample period | 2005-01-01 to 2022-12-31 |

**Comment 7**: The manuscript does not clearly explain how each article was mapped to a specific stock. What criteria were used — such as company name/entity recognition, ticker mentions, or other NLP-based rules? A brief methodological explanation is needed.
**Response**: We have provided a detailed explanation in response to Comment 5. Please refer to the response for Comment 5.

**Comment 8**: In Table 2, MV_MS holds fewer assets on average than MV_M, yet it incurs higher trading costs and turnover.
This result seems counterintuitive. Could the authors elaborate on why this is the case? A possible explanation might relate to more frequent or aggressive reallocations.
**Response**: This result suggests that the MV_MS model is more adaptive to market trends, leading to more frequent or substantial adjustments to its investment position. While it holds fewer assets on average, its strategy tends to reallocate more aggressively in response to changing market conditions. This results in higher trade costs and turnover rate compared to the MV_M.

**Comment 9**: Please check for any typos overall.
"abovementioned", "previou", "Secion", "prposed", "other assets set are as"
**Response**: Thank you for these corrections. We have thoroughly corrected and revised the manuscript.
